# Early Presentation of Boerhaave Syndrome in the Emergency Department: A Case Report and Review of the Literature

**DOI:** 10.3390/diagnostics14151592

**Published:** 2024-07-24

**Authors:** Irina-Anca Eremia, Cătălin-Alexandru Anghel, Florina-Alexandra Cofaru, Silvia Nica

**Affiliations:** 1 Emergency Department, “Carol Davila” University of Medicine and Pharmacy, 020021 Bucharest, Romania; irina.eremia@umfcd.ro (I.-A.E.); silvia.nica@umfcd.ro (S.N.); 2 Emergency Department, University Emergency Hospital Bucharest, 050098 Bucharest, Romania; florina.cofaru@drd.umfcd.ro

**Keywords:** esophageal rupture, Boerhaave syndrome, Meckler’s triad, vomiting effort, acute mediastinitis

## Abstract

We present the case of a 46-year-old patient who arrived at the emergency department with chest pain following an episode of vomiting. The diagnosis was Boerhaave syndrome and acute mediastinitis. Due to the prompt presentation and the location of the rupture in the lower esophagus, emergency surgical intervention was performed, including esophageal suturing, mediastinal drainage, and jejunostomy for feeding. Postoperatively, the patient was transferred to the intensive care unit for advanced monitoring and support. The patient’s condition improved favorably in the intensive care unit, allowing for extubation. Progress continued positively, and the patient was discharged 12 days postoperatively with a functional jejunostomy. At regular follow-ups, the patient’s recovery remained favorable, and the jejunostomy was removed 30 days after the surgery. This case highlights the importance of rapid diagnosis and early surgical intervention in Boerhaave syndrome, demonstrating the successful management of a severe condition through a multidisciplinary effort.

## 1. Introduction

Chest pain can be caused by conditions affecting the chest wall or intrathoracic viscera. The significance of pain in this area varies widely, ranging from minor, non-serious conditions to major medical emergencies that are life-threatening, where pain is the sole symptom. The presence of chest pain necessitates a differential diagnosis to determine its origin, which can be cardiac (ischemic myocardial pain, aortic pain, pericardial pain), pulmonary (tracheobronchial pain, pleural pain, diaphragmatic pain), gastrointestinal (esophageal spasm, esophageal rupture, peptic ulcer, esophagitis, cholecystitis, pancreatitis), or parietal (cutaneous pain, muscular pain, osteoarticular pain, radicular and neuralgic pain, mammary pain).

Spontaneous esophageal perforation (Boerhaave syndrome) was first described in 1724 by a professor of medicine at the University of Leiden. He reported the case of a high-ranking admiral of the Dutch fleet who, after a large meal, experienced pain in the left hemithorax accompanied by episodes of vomiting [1,2]. The patient died within 24 h [3]. Subsequently, the syndrome has been observed over time in individuals with tendencies toward excessive eating and drinking [4,5], and it has been recognized as a major surgical emergency. The prognosis of this condition depends significantly on the promptness of diagnosis and the initiation of treatment.

Boerhaave syndrome is a rare medical condition characterized by transmural esophageal perforation. The clinical presentation can be varied, and the entire process from the patient’s arrival in the emergency department to the appropriate treatment can be highly challenging. Mackler’s triad, characterized by chest pain, vomiting, and subcutaneous emphysema, is present in 50% of cases. As the patient exhibits more symptoms from this triad, the likelihood of diagnosis increases significantly [6,7,8]. Additionally, other atypical and nonspecific symptoms have been described, which can lead to further diagnostic errors: hematemesis [9,10], heartburn [10], acute hypoxemic respiratory failure [11], and abdominal tenderness [5,10,12]. In most cases, however, severe vomiting episodes are the cardinal symptoms of the clinical picture [13,14,15].

Spontaneous esophageal perforation accounts for approximately 15% of all esophageal rupture cases [4], while the majority of other cases are iatrogenic, most commonly occurring during upper gastrointestinal endoscopy [11,16]. Globally, the incidence is estimated to be around 3.1 per 1,000,000 per year, although this figure is thought to be underestimated [4,11]. This incidence was also reported in a national study conducted in Iceland [5,17]. The condition has been described across all racial groups, predominantly among men, with a male-to-female ratio ranging from 2:1 to 5:1. A significantly higher risk has been recorded among individuals aged 50 to 70 years [4,11], while the least affected age group is 1 to 17 years old [4].

The most commonly implicated risk factors are excessive alcohol consumption and overeating [11,18]. In addition to these, other etiological factors that have the potential to trigger the pathophysiological mechanism of Boerhaave syndrome should be considered: abdominal trauma, seizures, lifting weights, defecation, and not least, childbirth in women [4,11].

The most frequently cited cause in terms of the pathophysiological mechanism of Boerhaave syndrome is violent vomiting, which leads to a sudden increase in intraesophageal pressure due to both neuromuscular coordination failure between the lower and upper esophageal sphincters and the contracted cricopharyngeal muscle being unable to relax [4,10,19,20]. This increased intraesophageal pressure results in a complete transmural rupture of the distal esophageal wall in 80% of cases, typically located at the left posterior wall, approximately 3–6 cm above the diaphragm. This is attributed to several factors, including the anatomical discontinuity of the muscular layer, vascular and nervous anatomical elements penetrating the esophagus at this level, lesser adherence of other organs to the lower esophagus, inadequate supportive connective tissue surrounding it, forward inclination of the esophagus at the left diaphragmatic pillar [12], and structural differences in connective tissue between diagonal and circumferential fibers [4,10,14,19,20,21,22]. However, these factors remain theoretical, and the exact mechanism behind the condition is still uncertain [20]. The location of the rupture also plays a crucial role in understanding the patient’s clinical presentation and potential associated complications. Intrathoracic esophageal perforations, by allowing gastric contents into the adjacent spaces, can lead to mediastinal inflammation, emphysema, empyema, or necrosis. Perforations at the upper or middle part of the thoracic esophagus may be associated with hydropneumothorax or right pleural effusion [23,24,25].

Diagnosis can be confirmed in approximately 90% of cases with a contrast esophagogram, using a water-soluble contrast agent to avoid barium extravasation and its potential complications, such as inflammatory responses in mediastinal or pleural cavities [26]. The sensitivity of this method depends on factors characterizing the perforation (size and location) as well as the technique used. Studies have shown the possibility of false-negative results ranging from 10% to 38% [27]. In cases where examination with a water-soluble contrast agent cannot confirm the diagnosis but clinical suspicion remains high, a barium contrast esophagogram may be performed, which has a higher capacity to detect small perforations. Another diagnostic modality that can be utilized is contrast-enhanced CT (computed tomography) scan, which can provide additional details regarding the location of the rupture and drainable intrathoracic or intraabdominal collections. CT imaging may reveal thickening and edema of the esophageal wall, fluid in the pleural or retroperitoneal space, and widening of the mediastinum [4,27,28].

The key to the success of treating this condition lies in early diagnosis. The best prognosis is achieved for perforations diagnosed within the first 12–24 h. The approach to a patient with this pathology must be comprehensive, involving both medical and surgical aspects, provided by a multidisciplinary team comprising emergency medicine physicians, thoracic surgeons, gastroenterologists, and anesthesia and intensive care specialists. The therapeutic principles that should be applied at the initiation of treatment include avoidance of all oral intake, parenteral nutritional support, broad-spectrum antibiotic therapy, proton pump inhibitors, and drainage of collections or accumulated fluids [4]. Oral feeding can be resumed between the 7th and 14th day after radiological control. Regarding the management of perforations, these are repaired either surgically or endoscopically, depending on the location, size, the patient’s general condition, associated comorbidities, and importantly, the time elapsed from diagnosis to intervention.

Mortality in Boerhaave syndrome remains a significant challenge in current medical practice, reflecting the severity and complexity of this condition. Studies illustrate a high mortality rate [29], ranging between 20% and 75% [3], with variations depending on the severity of the rupture, its location, and the associated complications. In cases where diagnosis is delayed, mortality increases directly proportional to the number of hours lost from the onset of symptoms to elective interventions (some authors report rates as high as 40–60% [12] when treatment is initiated 48 h after symptom onset). Furthermore, untreated cases invariably lead to a mortality rate of 100% within a few days [3,30,31].

We chose to present this case because we believe that it is individualized by the classic clinical and paraclinical picture described in the specialized literature, which is why we want to sensitize clinicians to the importance of taking this possibility into account in establishing the differential diagnosis and not to miss it by simple omission. Also, the importance of this case is doubled by the fact that it is a spontaneous rupture of the esophagus (described in 15% of the cases in the specialized literature [2]).

## 2. Case

A 46-year-old patient, residing in an urban environment, presented to the emergency department (ED) following an episode of vomiting, accompanied by chest pain radiating to the cervical and epigastric regions. These symptoms started suddenly hours before presentation. Additionally, based on the medical history, the patient had no significant medical antecedents.

Physical examination at presentation revealed a patient with an altered general status, conscious, anxious, and psychomotor agitated (hemodynamically and respiratory stable). Clinical examination of the organ systems revealed the following pathological aspects: anxious face, pale, sweaty, cold, cyanotic extremities; muscular system—normotonic, normokinetic; osteoarticular system—clinically intact; respiratory system—tachypneic, dyspneic, thorax normally conformed, vesicular murmur present bilaterally, without added lung sounds, oxygen saturation measured by pulse oximetry = 98%; cardiovascular system—normal heart sounds, rhythmic, ventricular allure = 80 beats/min, without murmurs or added noises, peripheral pulse present bilaterally, blood pressure = 120/70 mmHg; digestive system—mobile with respiratory movements, antalgic contracture, difficult to examine; renal–urinary system—non-painful renal fossae; patient oriented temporally and spatially.

For the purpose of achieving a definitive diagnosis, the following investigations were performed in the emergency department (ED): complete blood count, biochemistry and coagulogram, blood group and Rh factor, EKG (sinus rhythm, without secondary repolarization changes), and CT examination with oral administration of contrast substance. 

The results of the blood tests upon the patient’s admission to the ED are summarized in Table 1.

**Figure 1:**
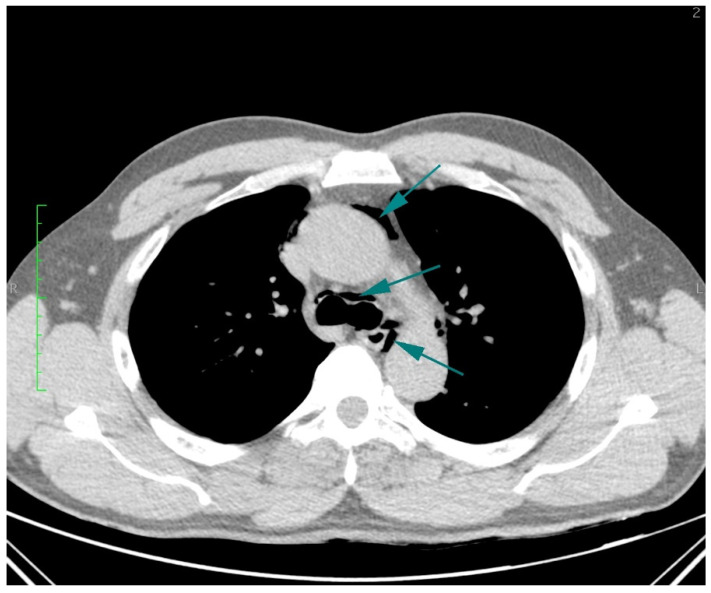
Transverse CT scan of the chest, demonstrating pneumomediastinum in moderate to large quantities (turquoise arrows), predominantly in the posterior mediastinum.

**Figure 2:**
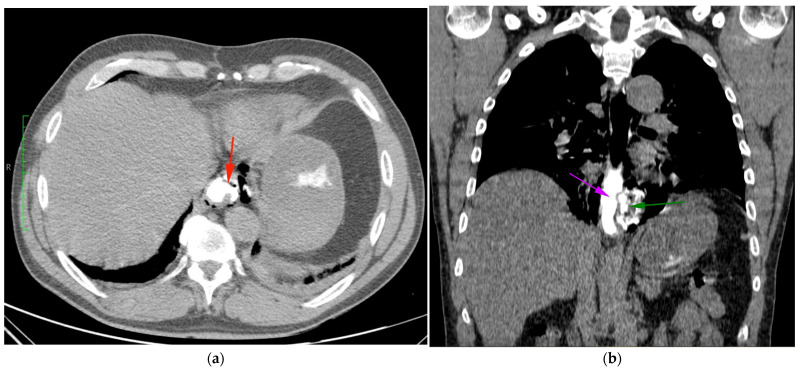
CT imaging post-oral contrast administration, highlighting extraluminal contrast extravasation into the mediastinum at the level of the distal esophagus: (**a**) transverse CT section: red arrow highlights the distal esophagus with thickened and edematous walls, and (**b**) longitudinal CT section: purple arrow indicates the perforated esophageal lumen through which the contrast substance is extravasated (green arrow).

**Figure 3:**
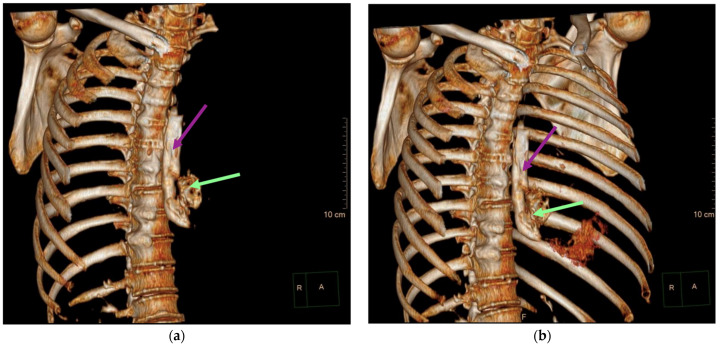
Three-dimensional reconstructions illustrating contrast extravasation into the mediastinum at the level of the distal esophagus: (**a**) 3D reconstruction showing the esophagus (purple arrow) and the extravasation of the contrast substance (green arrow), and (**b**) another perspective of the 3D reconstruction, highlighting the same findings.

**Figure 4:**
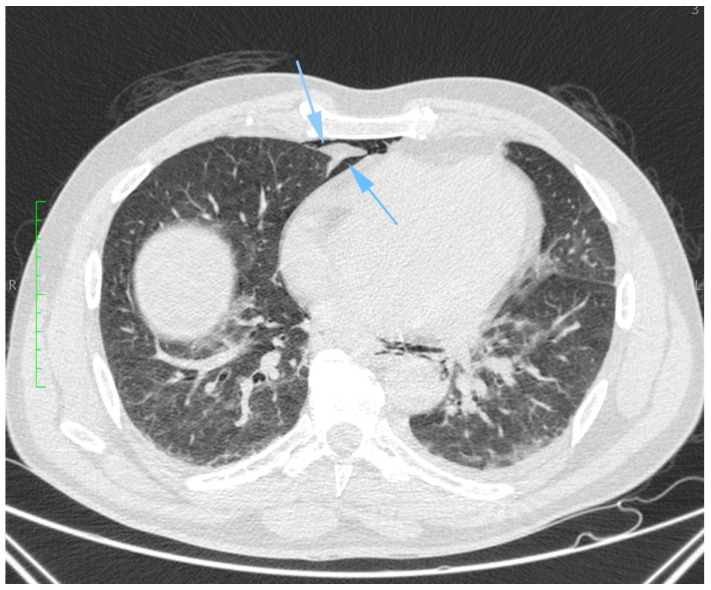
Transverse CT section, demonstrating a thin layer of right-sided pneumothorax (blue arrow).

Aortic dissection was the main diagnostic outcome considered based on the clinical and paraclinical data. Therefore, a decision was made to perform a native and contrast-enhanced CT examination of the thoraco-abdominal-pelvic region, which excluded this diagnosis and highlighted the following pathological findings: pneumomediastinum in moderate to large quantities, predominantly in the posterior mediastinum (Figure 1), with visualization of a fistulous tract at the level of the lower esophagus (lateral wall), the distal esophagus presenting thickened, edematous walls; after oral administration of the contrast agent, extravasation of the contrast product was observed extraluminally into the mediastinum at the level of the lower esophagus (Figure 2 and Figure 3), raising a suspicion of Boerhaave syndrome; fine right pneumothorax blade (Figure 4), distended stomach with liquid content.

Considering the clinical presentation and the results of the investigations conducted in the emergency department, the patient was admitted to the thoracic surgery department with diagnoses of Boerhaave syndrome and acute mediastinitis.

Given the patient’s presentation within the first hours of symptom onset and the localization of the rupture at the level of the lower esophagus, emergency surgical intervention was performed, including esophageal suturing, mediastinal drainage, and jejunostomy for feeding. Immediately postoperatively, the patient was transferred to the intensive care unit (ICU) for monitoring and advanced life support. Upon admission, the patient was continuously sedated with propofol, orotracheally intubated, and mechanically ventilated in controlled mode, hemodynamically stable, peripheral capillary oxygen saturation (SpO2) = 100%, blood pressure (BP) = 112/72 mmHg, heart rate (HR) = 98 bpm regular, with present diuresis and afebrile. A comprehensive bacteriological screening was performed, initiating broad-spectrum antibiotic therapy, antiemetic, antisecretory, anti-inflammatory, analgesic, and thromboprophylactic treatment, and hydroelectrolytic and acid–base balance therapy. The patient’s progression in the intensive care unit (ICU) was favorable; continuous sedation was stopped, and the patient became conscious and cooperative, leading to the decision to extubate. The patient remained on oxygen therapy via a face mask, with good gas exchange, hemodynamically stable, with present diuresis, and mildly febrile. It was decided to transfer the patient 24 h postoperatively to the thoracic surgery department for continued monitoring and specialized treatment. 

**Figure 5:**
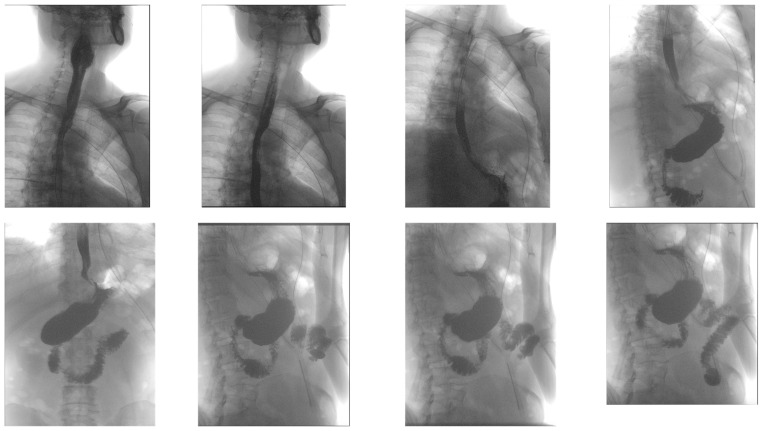
Sequence of images from the barium swallow examination 10 days postoperatively, showing an esophagus without fistulous tracts, no gastroesophageal reflux, no hiatal hernia, with patent cardia, normal-tone stomach, normal peristalsis with supple parallel folds, normal radiologic appearance of the bulb, pylorus, and duodenal frame, and no fistulous tracts at the feeding jejunostomy site.

**Figure 6:**
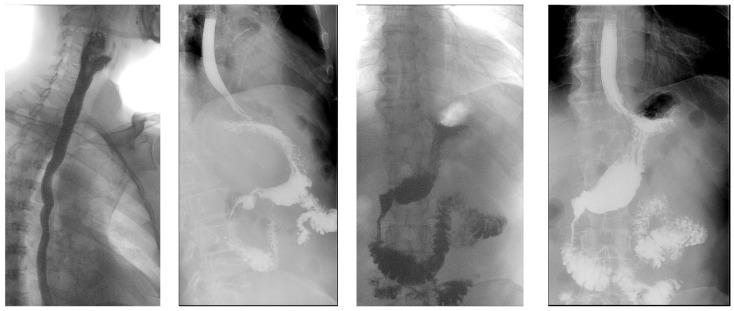
Sequence of images from the barium swallow examination at 60 days postoperative and at 30 days post-removal of the feeding jejunostomy, demonstrating a radiologically normal esophagus, no gastroesophageal reflux at the time of examination, no hiatal hernia, with a patent cardia, normal-tone stomach, normokinetic with supple, regular folds, patent pyloric orifice, normal radiologic appearance of the bulb and duodenal frame, normal morphology of intestinal loops, with slightly accelerated peristalsis.

Throughout the period of hospitalization, the patient’s progress was favorable, leading to discharge 12 days after the surgical intervention with a functional jejunostomy for feeding. Subsequently, he was called in for regular follow-up appointments, showing favorable progress, and the jejunostomy was removed 30 days after the surgical intervention (Figure 5 and Figure 6).

## 3. Discussion

Chest pain is one of the frequent symptoms with which people present to our emergency room and, undoubtedly, it can represent the clinical expression of a variety of pathologies, some of which are truly life-threatening emergencies. Although the most frequently implicated causes are cardiac and pulmonary [32], this case reminds us that there are other potentially lethal pathologies that we may very well overlook if we do not habitually include them in our differential diagnosis list. Among these is Boerhaave syndrome, which has a higher incidence among men in their sixth or seventh decade of life. In this instance, however, we were faced with a somewhat atypical presentation in a 46-year-old man, an age more suggestive of a cardiovascular condition, which was initially considered in the stage diagnosis due to its high probability. This was later ruled out, and the initial computed tomography (CT) images prompted the radiologist to administer oral contrast to verify the suspected diagnosis. Thus, the decision to perform a CT scan within the first hour of presentation allowed for a significant advancement in the diagnostic process, saving essential time and enabling early treatment initiation.

Another differential diagnosis that is often considered is Mallory–Weiss syndrome, characterized by longitudinal lacerations of the esophageal mucosa [5]. In this situation, the patient’s denial of hematemesis, instead reporting food vomiting without traces of blood, led us to consider from the outset that Mallory–Weiss syndrome was highly unlikely.

The term “spontaneous” that is often used to describe the esophageal rupture in Boerhaave syndrome is somewhat misleading, as in most cases, the esophageal rupture is the result of a sudden and extreme increase in intraesophageal pressure caused by vomiting. Moreover, this connection with violent vomiting is particularly relevant for oncology patients undergoing chemotherapy, where vomiting is a frequent side effect that affects their quality of life [33,34,35]. There are case reports describing oncology patients who developed Boerhaave syndrome as a result of chemotherapy-induced vomiting, including a case of a child with acute lymphoblastic leukemia [36] (the significance of this case also lies in the rarity of esophageal rupture in children) and a patient with hypopharyngeal cancer who developed the syndrome during chemoradiotherapy due to the combination of vomiting and hypopharyngeal stenosis [37]. This association underscores the need for careful management and continuous monitoring of oncology patients receiving chemotherapy. Prompt identification and treatment of severe vomiting and other relevant symptoms can reduce the risk of these potentially fatal complications.

The particularity of this case is also doubled by the early presentation of the patient in the emergency department, which allowed for the early restoration of esophageal continuity through suturing, resulting in an encouraging postoperative evolution and a very good long-term prognosis. In the management of this pathology, an alternative therapeutic option is represented by endoscopic therapy performed by an experienced endoscopist. The disadvantage of this method stems from observational studies in the literature that conclude a significant proportion of patients treated endoscopically require secondary intervention for various reasons [38,39]. An international study compared the outcomes of two groups of patients: one consisting of 20 patients treated with primary surgical intervention and another of 13 patients treated with endoscopic stenting. The results showed no significant differences in morbidity between the two groups. However, it was observed that 11 out of the 13 patients who received endoscopic therapy required reintervention [40]. In a systematic review that included 340 patients with esophageal perforation, endoscopic stenting demonstrated a technical success rate of 91.4% and a clinical success rate of 81.1%. Out of these, 58 patients (17%) required endoscopic reintervention due to persistent leaks, stent migration, or the need for stent repositioning. Additionally, 33 patients (10%) required surgical reintervention due to complications such as stent migration or perforation [41]. Further analysis highlights that despite the initial success in sealing the esophageal rupture with a stent, many patients subsequently developed complications such as pleural empyema or mediastinitis, necessitating additional surgical procedures. The failure rate of endoscopic stenting is significantly higher, with many cases eventually requiring surgical intervention to manage these complications effectively [40]. Moreover, the literature suggests that endoscopic stenting does not provide substantial advantages in terms of reducing the ICU stay, the hospital stay, or the prevalence of sepsis and organ failure compared to primary surgery [39,40]. Overall, while endoscopic therapy is a viable option, its limitations and high rate of secondary interventions necessitate careful patient selection to ensure optimal outcomes. For these reasons, we consider that endoscopic therapy should be reserved for selected cases that cannot benefit from surgical restoration of digestive continuity, including patients with multiple comorbidities or whose medical condition at the time of presentation does not permit surgical intervention. Additionally, the introduction of video-assisted thoracic surgery (VATS) offers more surgical options for Boerhaave syndrome. VATS involves the use of a thoracoscope and specialized instruments inserted through small incisions in the chest, allowing for a minimally invasive approach to thoracic surgery. This technique enables precise visualization and manipulation of thoracic structures, with reduced surgical trauma [42]. According to the study by Haveman et al., VATS has shown comparable results to open thoracotomy in terms of mediastinal and pleural cavity drainage and debridement, which are essential for managing Boerhaave syndrome. In the prospective group treated with VATS, only two of twelve cases required conversion to open thoracotomy, and the number of postoperative complications was reduced compared to the historical control group treated with open thoracotomy. These results suggest that VATS might be preferred due to the reduced morbidity and improved postoperative recovery for vulnerable patients [42]. Moreover, in this case, through adequate management and guided by the importance of a complete examination, a tragic outcome of a relatively rare pathology was avoided.

In the specialized literature, although there are no laboratory analyses with high sensitivity and specificity for diagnosing Boerhaave syndrome [4], certain changes in blood parameters have been observed in association with this condition. Among these, an increase in serum amylase levels can be explained by the pathophysiological mechanism of esophageal perforation, where saliva entering the bloodstream can lead to diagnostic errors, falsely suggesting acute pancreatitis. Therefore, it is crucial to carefully exclude the possibility of pancreatitis in such cases to avoid misdiagnosis.

Although diagnostic and treatment methods have significantly evolved from the late 19th century to the present, the mortality rate of patients with spontaneous esophageal rupture remains high, especially in cases of late diagnosis. The interventions available to improve the prognosis of these patients are often limited when the diagnosis is delayed. An article published in 1976 reported a postoperative survival rate of approximately 70% [30]. Today, postoperative survival rates have significantly improved, reflecting technological and therapeutic advances. However, these improvements are closely linked to the speed with which diagnosis and treatment are instituted, underscoring the importance of rapid intervention and continuous monitoring for patients with this severe condition.

## 4. Conclusions

Despite advances in imaging diagnosis and surgical management, mortality remains significant, underscoring the importance of early diagnosis and immediate surgical intervention in the management of these cases. Risk factors such as chronic alcoholism, uncontrolled gastroesophageal reflux, and hiatal hernia contribute to the complexity and severity of Boerhaave syndrome, highlighting the need for an integrated and multidisciplinary approach to improve the prognosis of affected patients.

The take-home message for every specialist working in primary care should be represented by the exhortation to never rule out a pathology based on only epidemiological probabilities because there are exceptional situations when the examined patient could even represent an exception.

## Figures and Tables

**Table 1:** The results of the laboratory blood tests.

Blood Test Category	Blood Test	Result	Reference Interval
Blood Count	White blood cell	15.09	4–10 × m/mm^3^
	Lymphocytes %	12.2	20–40%
	Monocytes %	9.5	3–10%
	Neutrophiles %	77.9	30–70%
	Eosinophiles %	0.0	0–7%
	Basophils %	0.4%	<1%
	Red blood cell %	5.56	3.8–6 M/mm^3^
	Mean corpuscular volume (MCV)	97.5	80–100 fL
	Mean corpuscular hemoglobin (MCH)	27.8	25–32 pg
	Mean corpuscular hemoglobin concentration (MCHC)	28.5	28–36 g/dL
	Hematocrit	54.2	33–54%
	Hemoglobin	15.5	10–16.5 g/dL
	Platelet count	408	100–450 m/mm^3^
	Mean platelet volume (MPV)	7.9	6–13 fL
Biochemistry Tests	Glucose	173	74–106 mg/dL
	Urea	40.9	17–43 mg/dL
	Creatinine	1.39	0.67–1.17 mg/dL
	Alanine aminotransferase (ALT)	72	0–50 U/L
	Aspartate aminotransferase (AST)	97	0–50 U/L
	Bilirubin total	0.52	0.3–1.2 mg/dL
	Bilirubin direct	0.09	0–0.2 mg/dL
	Amylase	155	28–100 U/L
	Lipase	87	8–78 U/L
	Creatine kinase (CK)	6480	0–171 U/L
	Creatine kinase MB (CK-MB)	96	0–25 U/L
	Sodium	142	136–146 mmol/L
	Potassium	3.58	3.5–5.1 mmol/L
	hs-cTnI	18	<28.9 ng/L
Coagulogram	Prothrombin time (PT)	12.9	9.4–12.5 s
	INR	1.17	0.800–1.140
	Activated partial thromboplastin time (aPTT)	22.7	22.0–36.0 s
	Fibrinogen	351	238–498 mg/dL

## Data Availability

This article does not include any additional primary data besides the information already presented in the case report section.
